# The additive effects of obesity on myocardial microcirculation in diabetic individuals: a cardiac magnetic resonance first-pass perfusion study

**DOI:** 10.1186/s12933-020-01028-1

**Published:** 2020-05-06

**Authors:** Li Jiang, Ke Shi, Ying-kun Guo, Yan Ren, Zhen-lin Li, Chun-chao Xia, Lei Li, Xi Liu, Lin-jun Xie, Yue Gao, Meng-ting Shen, Ming-yan Deng, Zhi-gang Yang

**Affiliations:** 1 Department of Radiology, West China Hospital, Sichuan University, 37# Guo Xue Xiang, Chengdu, Sichuan 610041 China; 2 Department of Radiology, Key Laboratory of Birth Defects and Related Diseases of Women and Children of Ministry of Education, West China Second University Hospital, Sichuan University, 20# South Renmin Road, Chengdu, Sichuan 610041 China; 3 Department of Endocrinology and Metabolism, West China Hospital, Sichuan University, 37# Guo Xue Xiang, Chengdu, Sichuan 610041 China

**Keywords:** Body mass index, Cardiac magnetic resonance first-pass perfusion imaging, Myocardial microvascular function, Obesity, Type 2 diabetes mellitus

## Abstract

**Background:**

The microvascular effects of obesity should be considered in diabetic individuals for elucidating underlying mechanisms and developing targeted therapies. This study aims to determine the effect of obesity on myocardial microvascular function in type 2 diabetes mellitus (T2DM) patients using cardiac magnetic resonance (CMR) first-pass perfusion imaging and assessed significant risk factors for microvascular dysfunction.

**Materials and methods:**

Between September 2016 and May 2018, 120 patients with T2DM (45.8% women [55 of 120]; mean age, 56.45 ± 11.97 years) and 79 controls (44.3% women [35 of 79]; mean age, 54.50 ± 7.79 years) with different body mass index (BMI) scales were prospectively enrolled and underwent CMR examination. CMR-derived perfusion parameters, including upslope, time to maximum signal intensity (TTM), maximum signal intensity (MaxSI), MaxSI (-baseline), and SI (baseline), and T2DM related risk factors were analyzed among groups/subgroups both in T2DM patients and controls. Univariable and multivariable linear and logistic regression analyses were performed to assess the potential additive effect of obesity on microvascular dysfunction in diabetic individuals.

**Results:**

Compared with controls with comparable BMIs, patients with T2DM showed reduced upslope and MaxSI and increased TTM. For both T2DM and control subgroups, perfusion function gradually declined with increasing BMI, which was confirmed by all perfusion parameters, except for TTM (all *P *< 0.01). In multivariable linear regression analysis, BMI (β = − 0.516; 95% confidence interval [CI], − 0.632 to − 0.357; *P *< 0.001), female sex (β = 0.372; 95% CI, 0.215 to 0.475; *P *< 0.001), diabetes duration (β = − 0.169; 95% CI, − 0.319 to − 0.025; *P *= 0.022) and glycated haemoglobin (β = − 0.184; 95% CI, − 0.281 to − 0.039; *P *= 0.010) were significantly associated with global upslope in the T2DM group. Multivariable logistic regression analysis indicated that T2DM was an independent predictor of microvascular dysfunction in normal-weight (odds ratio[OR], 6.46; 95% CI, 2.08 to 20.10; *P *= 0.001), overweight (OR, 7.19; 95% CI, 1.67 to 31.07; *P *= 0.008) and obese participants (OR, 11.21; 95% CI, 2.38 to 52.75; *P *= 0.002).

**Conclusions:**

Myocardial microvascular function gradually declined with increasing BMI in both diabetes and non-diabetes status. T2DM was associated with an increased risk of microvascular dysfunction, and obesity exacerbated the adverse effect of T2DM.

## Background

Diabetes mellitus (DM) and obesity, which are common chronic diseases that often coexist, impact millions of individuals worldwide and are ﻿contributors to the worsening global health burden [1, 2]. Both DM and obesity are associated with an increased overall risk of premature death due to systemic complications, and cardiovascular disease is the primary complication and leading cause of death [3–5]. Microvascular myocardial dysfunction has recently emerged as an additional mechanism of myocardial impairment that has important prognostic implications [6, 7]. However, several studies investigating the relationship of microvascular function with DM or obesity have focused on the effects of DM and obesity as separate entities, and the contribution of obesity to myocardial microvascular dysfunction in DM is not fully understood and clear [8–10]. Elucidation of the interaction between DM and obesity can aid in the understanding of mechanisms underlying the development of microvascular dysfunction and in the identification of patient subgroups that can benefit from targeted therapies.

Cardiac magnetic resonance (CMR)-derived first-pass perfusion imaging is a promising technique to assess myocardial perfusion due to its high spatial and temporal resolution, and has been increasingly utilised for the non-invasive evaluation of myocardial microvascular function [11, 12]. Therefore, we aimed to determine the effect of obesity on myocardial microvascular function in type 2 DM (T2DM) patients using CMR first-pass perfusion imaging and explored significant risk factors contributing to microvascular dysfunction in patients with T2DM.

## Materials and methods

### Study population

**Fig. 1:**
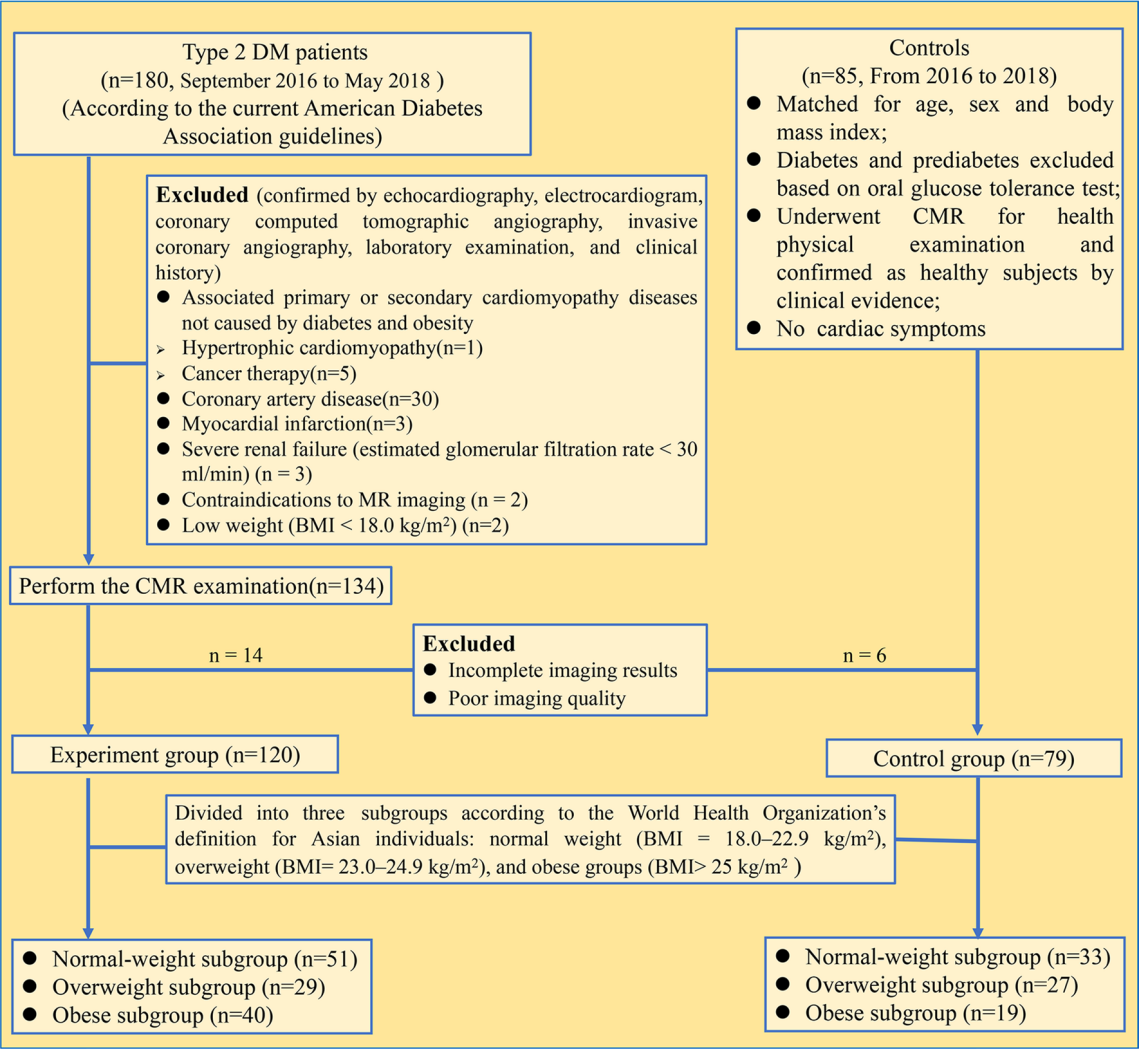
Flow diagram of the cohort study. Of the consecutive 180 patients with T2DM who were enrolled, 46 were excluded before CMR examination, and 14 were excluded after CMR examination owing non-available perfusion images. *MaxSI* maximum signal intensity, *SI* signal intensity, *TTM* time to maximum signal intensity

﻿Between September 2016 and May 2018, 180 consecutive patients with T2DM were prospectively enrolled in our study and eligible for CMR examination. The inclusion criteria were clinically confirmed subjects with T2DM in the endocrinology department of our hospital according to the current American Diabetes Association criteria [13]. Detailed exclusion criteria are shown in Fig. 1, including associated primary or secondary cardiomyopathy diseases not caused by diabetes and obesity [14] (n = 6), coronary artery disease (n = 30), myocardial infarction (n = 3), incomplete CMR perfusion examination (n = 5), low weight (body mass index [BMI] < 18.0 kg/m^2^) (n = 2), poor CMR image quality (n = 3), and unavailable perfusion parameters (n = 11). Therefore, 120 T2DM patients (45.8% women [55 of 120]; mean age, 56.45 ± 11.97 years) were finally included. During the study period, 79 age-, sex-, and BMI-matched individuals (44.3% women [35 of 79]; mean age, 54.50 ± 7.79 years) were included as controls who underwent CMR for health physical examination. The aforementioned exclusion criteria for the T2DM group also applied to the control group. Moreover, all included controls performed the ﻿oral glucose tolerance test to rule out possible diabetes and prediabetes in the endocrinology department of our hospital. The participants were divided into the following weight groups according to the World Health Organization’s definition for Asian individuals [15, 16]: normal weight (BMI = 18.0–22.9 kg/m^2^), overweight (BMI = 23.0–24.9 kg/m^2^), and obese groups (BMI > 25 kg/m^2^). Related clinical and imaging variables were collected for all participants.

The Biomedical Research Ethics Committee of our hospital approved this study, and all participants gave written informed consent. The participant-sensitive information was protected with full confidentiality and was only used for the purposes of this study.

### CMR protocol

All participants were examined using a 3.0T whole-body scanner (Trio Tim; Siemens Medical Solutions, Erlangen, Germany) in the supine position at rest. The manufacturer’s standard ECG-triggering device and the breath-hold technique were used to monitor dynamic changes in each participant’s ECG findings and breathing. Cine images were acquired in two-chamber, three-chamber, four-chamber, and short-axis views using a steady-state free precession sequence (repetition time[TR]/echo time[TE], 33.22/1.31 ms; flip angle, 39°; slice thickness, 8.0 mm; field of view, 234 × 280 mm^2^; matrix size, 208 × 139). A bolus of gadobenate dimeglumine (MultiHance; 0.5 mmol/ml; Bracco, Milan, Italy) was intravenously injected at a dose of 0.2 ml/kg body weight and a flow rate of 2.5– 3.0 ml/s. A 20-ml saline flush was administered immediately following contrast at a rate of 3.0 ml/s. First-pass perfusion was acquired concurrent with intravenous contrast agents in three standard short-axis slices (the basal, mid-ventricular, and apical slices) and performed by inversion recovery prepared echo-planar sequence (TR/TE, 200/1.1 ms; flip angle, 10°; field of view, 270 × 360 mm^2^; matrix size, 106 × 192). Each set of first-pass perfusion images was acquired in 80 cardiac cycles. Each participant’s condition was stable and feasible during the entire examination period.

### CMR image analysis

**Fig. 2:**
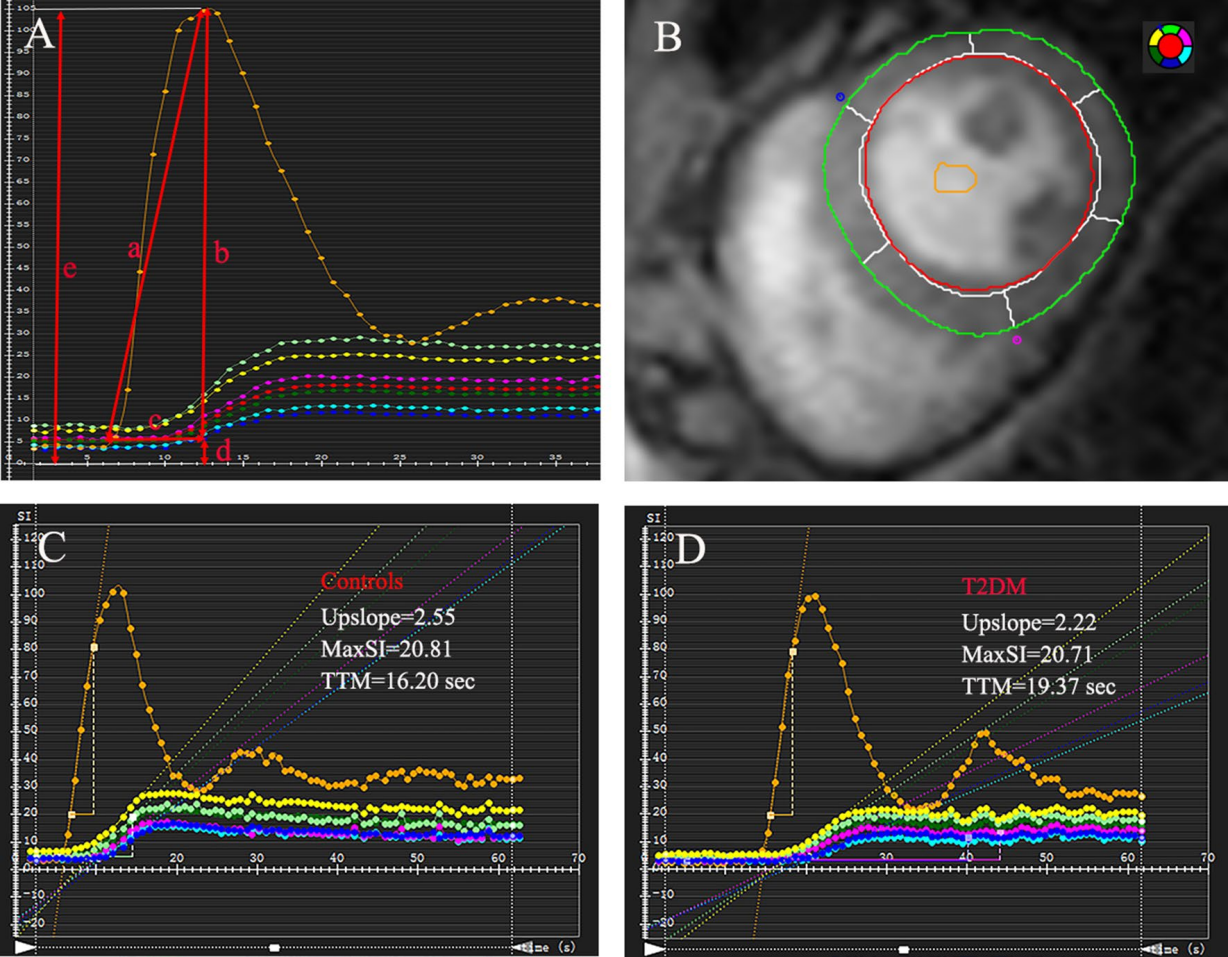
CMR-derived perfusion image analysis. The first-pass perfusion parameters including upslope (**a**), MaxSI (-baseline) (**b**), TTM (**c**), MaxSI (**e**), and SI (baseline) (**d**) were automatically obtained from time–signal intensity (**A**); Representative myocardial and blood-pooled time–signal intensity curve obtained from left ventricle mid-ventricular slices (**B**) showed reduced upslope and MaxSI and increased TTM in patient with diabetes (**D**) compared to control (**C**)

All images of the patients and controls were transferred to offline commercial software (CVI^42^; Circle Cardiovascular Imaging, Inc., Calgary, Canada) and were analyzed by two experienced radiologists. For regional analysis, a 16-segment model (Bull’s eye plot) was constructed on the basis of three standard short-axis slices, and the endocardial and epicardial borders of all three slices of first-pass perfusion images were delineated manually, with exclusion of papillary muscles and trabeculations, and a region of interest was drawn in the blood pool as contrast (Fig. 2b). The myocardial and blood-pooled time–signal intensity curve was obtained for each myocardial segment in all participants (Fig. 2c, d).

CMR-derived perfusion parameters, including upslope, time to maximum signal intensity (TTM), maximum signal intensity (MaxSI), MaxSI (-baseline), and SI (baseline) were obtained from the myocardial time–signal intensity just as in Fig. 2a. In the three short-axis slices, the average perfusion value for each slice was automatically calculated by the software. The global upslope was calculated as the average parameter value of 16 segments in this study. All parameters reflected myocardial perfusion, which is indirectly related to coronary microvascular function [8].

LV myocardial mass (LVM) and LV functional parameters, including LV end-diastolic volume (LVEDV), LV end-systolic volume (LVESV), and LV ejection fraction (LVEF), were derived from CMR cine images according to current guidelines [17]. In addition, the LV remodeling index was applied and calculated as “LVM/LVEDV” [18].

### Assessment of the reproducibility of perfusion parameters

To assess intra-observer reproducibility, a radiologist (JL) drew endocardial and epicardial borders in three slices of the first-pass perfusion images in 60 randomly selected participants, including patients with T2DM and controls, in two sessions separated by 2 months. To assess inter-observer variability of the perfusion parameters, another radiologist (SK) repeated the data processing in 55 randomly selected participants, including patients with T2DM and controls. Each radiologist was blinded to the subject status (T2DM or control) and the findings of the other radiologist during the variability assessment.

### Statistical analysis

The continuous normally distributed variables are expressed as mean ± standard deviation using Student’s *t* test, whereas non-normally distributed variables were presented as median and inter-quartile range using the Mann–Whitney test. The categorical variables are expressed as number (percentage), and were compared using Fisher’s exact test. LV myocardial perfusion parameters and T2DM related risk factors were compared between DM patients and controls using the Student’s t-test and were compared among subgroups across different BMI scales using one-way analysis of variance (ANOVA) followed by Bonferroni’s post hoc test. Univariable linear regression analysis was performed to identify the predictors of reduced global upslope in patients with T2DM. All candidate variables for multivariable models were selected based on clinical grounds, guided by univariable analysis with P value < 0.1 and ﻿the absence of collinearity. The receiver operator characteristic (ROC) curve for global upslope was generated to discriminate the additive effect of the T2DM status in controls with similar BMIs, and the optimal cut-off value based on the ROC curve was used to classify abnormal and normal microcirculation groups within the same BMI scale, wherein only the additive effect of T2DM on microcirculation was considered and the effect of BMI was classified as the normal group. Further logistic regression analysis was performed to determine the effect of T2DM status on myocardial microvascular function in individuals with different BMI scales. A *P* value of < 0.05 was regarded to indicate statistical significance. All analyses were performed using SPSS (version 24.0; IBM, Armonk, NY, USA) and GraphPad Prism^®^ (version 7.0a; GraphPad Software, San Diego, CA, USA).

## Results

### Baseline characteristics

The study cohort comprised 120 patients with T2DM, including 51, 29 and 40 normal-weight, overweight and obese individuals, respectively, and 79 nondiabetic controls, including 33, 27 and 19 normal-weight, overweight and obese individuals. The baseline characteristics of the nondiabetic controls and patients with T2DM in different BMI scales (normal-weight, overweight and obese groups) are presented in Table 1. There were no significant statistical differences in age, sex and BMI among the subgroups, however, the diabetes subgroup presented with higher systolic blood pressure than the control subgroup with the same BMI scales. Importantly, among the cardiac morphological parameters, the left ventricular (LV) remodelling index gradually increased with increasing BMI in both the T2DM and control groups (*P* < 0.05) and the mean LV remodeling index was higher in the T2DM group than in the control group.

**Table 1:** Baseline characteristics of the controls and DM patients

	Controls (n = 79)				DM patients (n = 120)				P value^∂^	P value^‡^	P value^∆^
	Normal-weight (n = 33)	Overweight (n = 27)	Obese (n = 19)	P value^å^	Normal-weight (n = 51)	Overweight (n = 29)	Obese (n = 40)	P value^ß^			
Age (y)	55.27 ± 8.18	54.59 ± 9.12	56.89 ± 9.79	0.462	55.06 ± 13.21	58.00 ± 10.05	57.00 ± 11.09	0.541	0.935	0.202	0.972
Female	13 (39.3%)	13 (29.6%)	9 (42.1%)	0.680	28 (54.9%)	10 (27.6%)	17 (35.0%)	0.577	0.186	0.416	0.784
BMI (kg/m^2^)	21.94 ± 1.01	24.00 ± 0.55*	26.53 ± 0.90* ^§^	0.000	21.26 ± 1.21	23.75 ± 0.56 *	26.92 ± 1.59 * ^§^	0.000	0.073	0.114	0.227
Smoking history	8 (24.2%)	8 (29.6%)	7 (36.8%)	0.627	9 (17.6%)	8 (27.6%)	10 (25.0%)	0.532	0.580	0.550	0.372
Diabetes duration (y)	–	–	–	–	6.51 ± 4.89	9.28 ± 6.51	9.35 ± 6.11	0.033	–	–	–
SBP (mm Hg)	121.0 ± 11.7	119.7 ± 14.2	121.0 ± 13.1	0.917	127.8 ± 16.9	132.9 ± 22.3	129.1 ± 14.4	0.515	0.026	0.017	0.071
DBP (mm Hg)	77.0 ± 8.8	78.7 ± 9.5	73.6 ± 9.8	0.197	81.3 ± 12.7	79.5 ± 10.0	79.4 ± 9.7	0.748	0.101	0.743	0.052
HbA1c (%)	5.32 ± 0.44	5.41 ± 0.51	5.38 ± 0.48	0.815	7.9 ± 2.4	7.8 ± 2.2	7.9 ± 1.6	0.944	0.000	0.000	0.000
TG (mmol/L)	1.32 ± 0.57	1.85 ± 1.25	2.12 ± 1.55	0.034	1.59 ± 1.34	1.54 ± 0.75	2.39 ± 2.87	0.090	0.278	0.262	0.706
TC (mmol/L)	4.21 ± 0.95	4.51 ± 0.96	4.98 ± 1.52	0.535	4.36 ± 1.18	4.18 ± 0.71	4.36 ± 1.25	0.759	0.869	0.496	0.463
HDL (mmol/L)	1.34 ± 0.14	1.38 ± 0.52	1.42 ± 0.27	0.727	1.48 ± 0.50	1.18 ± 0.33	1.30 ± 01.01*	0.153	0.122	0.091	0.616
LDL (mmol/L)	2.52 ± 0.49	2.49 ± 0.77	2.29 ± 0.85	0.485	2.58 ± 0.85	2.41 ± 0.59	2.39 ± 0.77	0.453	0.738	0.656	0.650
eGFR (mL/min)	95.39 ± 9.39	98.76 ± 11.14	98.28 ± 8.72	0.371	89.66 ± 21.41	85.81 ± 24.33	87.30 ± 25.72	0.766	0.097	0.013	0.019
Cardiac troponin I (ug/L) -		–	–	–	0.025 ± 0.023	0.022 ± 0.009	0.029 ± 0.013	0.197	–	–	–
Atrial fibrillation	–	–	–	–	0	1 (3.4%)	1 (2.5%)	0.450	–	–	–
Renal dysfunction^	–	–	–	–	8 (15.7%)	6 (20.7%)	9 (22.5%)	0.695	–	–	–
Retinopathy	–	–	–	–	4 (7.8%)	3 (10.3%)	6 (15.0%)	0.549	–	–	–
Peripheral neuropathy	–	–	–	–	13 (25.5%)	10 (34.5%)	18 (45.0%)	0.124	–	–	–
Biguanides	–	–	–	–	18 (35.3%)	13 (44.8%)	21 (52.5%)	0.254	–	–	–
Sulfonylureas	–	–	–	–	9 (17.6%)	4 (13.7%)	10 (25.0%)	0.474	–	–	–
α-Glucosidase inhibitor	–	–	–	–	13 (25.5%)	11 (37.9%)	18 (45.0%)	0.140	–	–	–
GLP-1/DPP-4 inhibitor	–	–	–	–	3 (5.9%)	2 (6.9%)	2 (5.0%)	0.946	–	–	–
Insulin	–	–	–	–	12 (23.5%)	8 (27.6%)	6 (15.0%)	0.417	–	–	–
ACEI/ARB	–	–	–	–	9 (17.6%)	6 (20.7%)	8 (20.0%)	0.934	–	–	–
Statin	–	–	–	–	4 (7.8%)	7 (24.1%)	8 (20.0%)	0.107	–	–	–
LVEDV/BSA (ml/m^2^)	73.53 ± 13.54	78.71 ± 17.55	76.30 ± 13.77	0.418	80.21 ± 24.54	80.56 ± 20.30	70.31 ± 12.41	0.040	0.157	0.717	0.100
LVESV/BSA (ml/m^2^)	29.89 ± 11.68	32.37 ± 14.67	33.18 ± 18.51	0.685	32.98 ± 18.77	32.71 ± 16.69	29.33 ± 10.81	0.519	0.400	0.937	0.318
LVEF (%)	61.68 ± 4.82	60.52 ± 8.11	61.86 ± 5.71	0.718	60.11 ± 7.75	61.39 ± 12.09	59.64 ± 9.04	0.738	0.301	0.755	0.343
LV mass/BSA (g/m^2^)	47.26 ± 15.33	49.75 ± 18.76	50.87 ± 10.37	0.685	52.62 ± 19.41	56.93 ± 13.55	52.67 ± 14.15	0.479	0.185	0.105	0.617
Rest heart rate	70.6 ± 11.4	73.5 ± 11.9	72.5 ± 9.0	0.585	74.9 ± 12.1	71.4 ± 10.0	73.4 ± 9.4	0.396	0.110	0.483	0.732
LV remodeling index^#^	0.59 ± 0.11	0.64 ± 0.13	0.69 ± 0.16	0.047	0.66 ± 0.14	0.72 ± 0.13	0.75 ± 0.15*	0.008	0.020	0.046	0.157

Values are means ± standard deviations or n (%)

P value^å^, P value^**ß**^: P values refer to the differences among three subgroups across different BMI scales within controls (^å^) or DM patients (^ß^) based on Fisher’s exact test or one-way analysis of variance followed by Bonferroni’s post hoc test; * p < 0.017 versus Normal weight group; ^§^p < 0.017 versus overweight group

P value^**∂**^, P value^**‡**^ and P value^∆^: DM patients versus controls based on Fisher’s exact test or Student’s t-test in normal-weight (P value^**∂**^), overweight (P value^**‡**^), and obese (P value^∆^) subgroups, respectively

**^**Renal dysfunction: estimated creatinine clearance < 60 mL/min; ^#^LV remodeling index: LV mass/LVEDV. *BMI* body mass index, *BSA* body surface area, *eGFR* estimated glomerular filtration rate, *SBP* systolic blood pressure, *DBP* diastolic blood pressure, *TG* plasma triglycerides, *TC* total cholesterol, *HDL* high-density lipoprotein, *ACEI* asngiotensin converting enzyme inhibitor, *ARB* angiotensin receptor blockers

### Comparison of the microvascular function between the T2DM and control groups

**Fig. 3:**
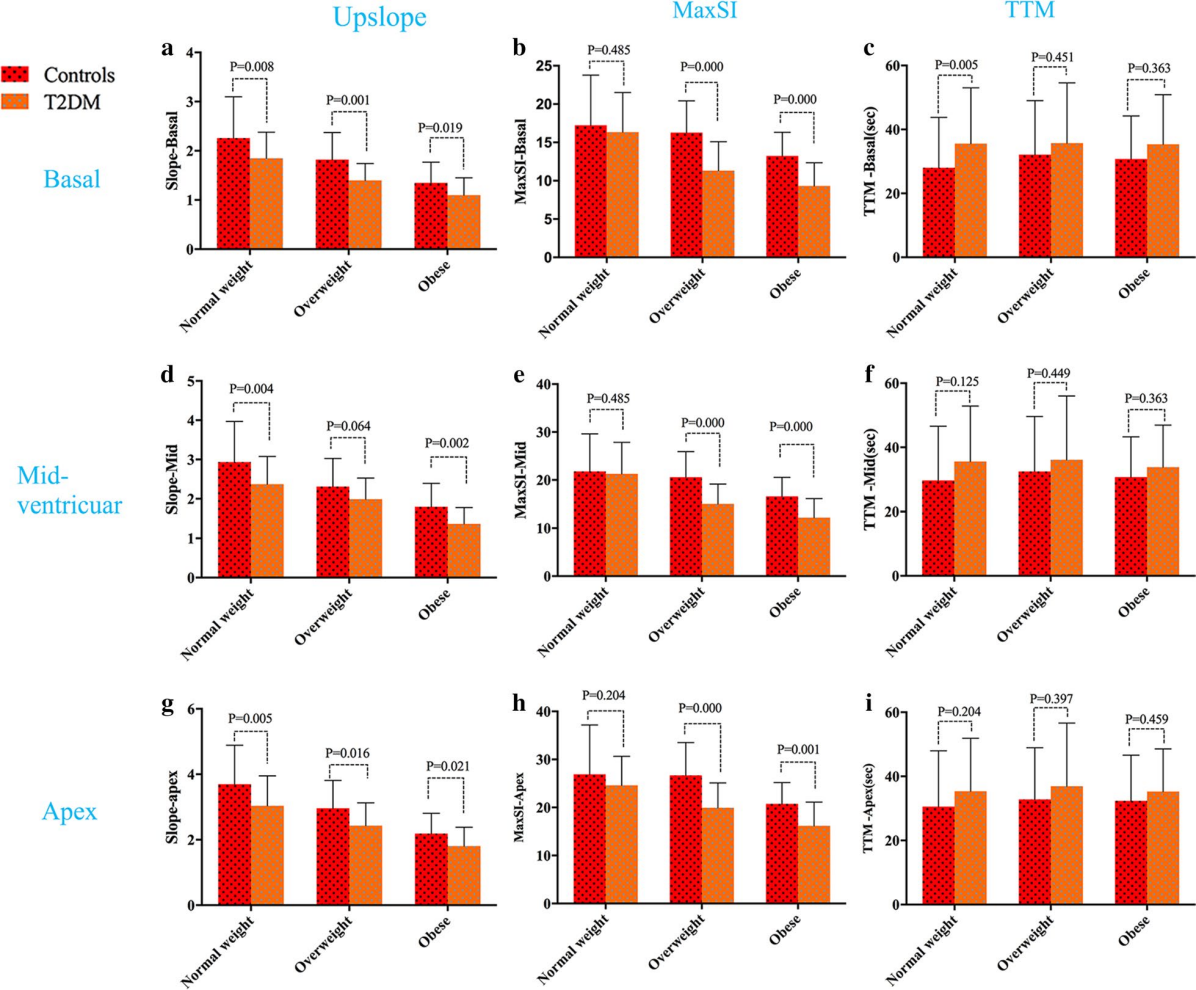
The perfusion function between T2DM and controls subgroup on the same weight scale. T2DM patients showed significantly worse microvascular function in all perfusion parameters when compared with controls on the same weight scale in three standard short-axis slices (basal, mid-ventricular and apex slice). MaxSI, maximum signal intensity; TTM, time to maximum signal intensity

On comparing the T2DM and controls on the same BMI scale, the T2DM patients had worse microvascular perfusion function in the three short-axis slices, which was supported by reduced upslope, reduced MaxSI, and increased TTM values in T2DM patients, respectively (Fig. 3).

Furthermore, the three short-axis slices involved different perfusion functions, and the upslope, MaxSI and SI (baseline) values increased gradually from the base to the apex in all participants. Nevertheless, there was no significant change in the TTM from the base to the apex.

### Microvascular function gradually decreases with increasing BMI in all patients

The microvascular perfusion parameters of the controls and patients with T2DM according to the different BMI scales are presented in Table 2. Briefly, microvascular function gradually decreased with increasing BMI in both the patients with T2DM and the controls, which was evident by all perfusion parameters with the exception of TTM (*P *< 0.05 for all). Bonferroni’s post hoc test revealed that the multi-parameter (upslope, MaxSI and MaxSI (-BL)) exhibited a statistically significant difference between the obese and normal-weight subgroups in both the control and T2DM groups (*P* < 0.017). Moreover, multiple perfusion parameters (upslope, MaxSI and MaxSI (-BL)) were significantly different between the overweight and normal-weight subgroups among those with T2DM but not among the controls (*P* < 0.017 for all).

**Table 2:** Perfusion parameter of controls (n = 79) and DM patients (n = 120) with different BMI

	Controls (n = 79)					DM patients (n = 120)			
	Normal-weight (n = 33)	Overweight (n = 27)	Obese (n = 19)	P value^**å**^		Normal-weight (n = 51)	Overweight (n = 29)	Obese (n = 40)	P value^**ß**^
Basal									
Upslope	2.26 ± 0.84	1.82 ± 0.55	1.35 ± 0.42*	0.000		1.85 ± 0.53	1.40 ± 0.34*	1.10 ± 0.35* ^§^	0.000
TTM (sec)	25.22 ± 10.85	32.17 ± 17.42	33.17 ± 14.12	0.084		35.36 ± 17.05	35.75 ± 18.83	35.36 ± 15.52	0.929
MaxSI	17.24 ± 6.54	16.27 ± 4.14	13.24 ± 3.07*	0.028		16.34 ± 5.18	11.33 ± 3.76*	9.33 ± 3.01*	0.000
MaxSI (- baseline)	15.93 ± 4.26	13.29 ± 4.61	10.62 ± 2.65*	0.000		12.63 ± 4.40	8.06 ± 2.58*	7.31 ± 2.52*	0.000
SI (baseline)	3.72 ± 1.43	3.35 ± 0.95	2.63 ± 0.80*	0.006		3.13 ± 1.42	2.29 ± 0.87*	2.20 ± 0.95*	0.000
Mid-ventricular									
Upslope	2.93 ± 1.03	2.31 ± 0.72*	1.80 ± 0.60*		0.000	2.37 ± 0.70	1.99 ± 0.54*	1.37 ± 0.41* ^§^	0.000
TTM (sec)	27.31 ± 13.86	31.68 ± 16.61	33.25 ± 13.83		0.313	34.19 ± 16.14	35.93 ± 20.13	34.26 ± 12.55	0.884
MaxSI	21.78 ± 7.83	20.59 ± 5.32	16.58 ± 3.96*		0.018	21.29 ± 6.54	15.01 ± 4.14*	12.22 ± 3.91*	0.000
MaxSI (- baseline)	19.63 ± 5.25	16.74 ± 5.41	13.14 ± 3.42*		0.000	16.38 ± 5.39	10.83 ± 3.48*	9.49 ± 3.33*	0.000
SI (Baseline)	4.83 ± 1.60	4.36 ± 1.51	3.39 ± 0.96*		0.004	4.22 ± 1.73	3.08 ± 1.00*	2.86 ± 1.06*	0.000
Apex									
Upslope	3.69 ± 1.19	2.95 ± 0.85*	2.19 ± 0.62*		0.000	3.03 ± 0.91	2.44 ± 0.70*	1.80 ± 0.57* ^§^	0.000
TTM (sec)	27.81 ± 13.87	32.43 ± 16.55	35.01 ± 14.62		0.221	34.20 ± 15.75	36.74 ± 19.94	35.26 ± 13.31	0.794
MaxSI	26.90 ± 10.28	26.67 ± 6.84	20.76 ± 4.42		0.021	24.42 ± 8.50	19.94 ± 5.17*	16.19 ± 4.91*	0.000
MaxSI (- baseline)	24.32 ± 7.72	21.37 ± 5.41	16.50 ± 4.03*		0.000	21.52 ± 7.40	13.82 ± 4.21*	12.68 ± 4.10*	0.000
SI (baseline)	5.80 ± 2.06	5.27 ± 2.05	4.36 ± 1.47		0.041	5.57 ± 2.16	4.15 ± 1.33*	3.70 ± 1.38*	0.000

Values are means ± standard deviations

P value^å^, P value^**ß**^: P values refer to the differences among three subgroups across different BMI scales within controls (^å^) or DM patients (^ß^) based on Fisher’s exact test or one-way analysis of variance followed by Bonferroni’s post hoc test; *P < 0.017 versus Normal weight group; ^§^P < 0.017 versus overweight group

### The linear regression analysis of microvascular function in all T2DM patients

As shown in Table 3, age, sex, BMI, diabetes duration, ﻿glycated haemoglobin (HbA1c), plasma triglycerides, systolic blood pressure, estimated glomerular filtration rate, LV remodelling index and medication history were initially screened based on clinical grounds and assessed using univariable analysis. The multivariable analysis model (R^2^ = 0.567) showed that female sex had a positive microcirculation effect (β = 0.372; 95% confidence interval[CI], 0.215 to 0.475; *P *< 0.001) and that BMI (β = − 0.516; 95% CI, − 0.632 to − 0.357; *P *< 0.001), diabetes duration (β = − 0.169; 95% CI, − 0.319 to −0.025; *P *= 0.022) and HbA1c (β = − 0.184; 95% CI, − 0.281 to − 0.039; *P *= 0.010) had a negative microcirculation effect.

**Table 3:** Univariable and multivariable linear regression analysis of global upslope in all DM patients (n = 120)

	Univariable		Multivariable^a^		
	β	P value	β	P value	R^2^
Age (y)^∆^	− 0.048	0.501	–	–	0.567
Sex (female)	0.351	0.000	0.372	0.000	
BMI (kg/m^2^)	− 0.581	0.000	− 0.516	0.000	
Diabetes duration (year)	− 0.347	0.000	− 0.169	0.022	
HbA1c (%)	− 0.314	0.000	− 0.184	0.010	
TG (mmol/L)	− 0.153	0.031	–	–	
SBP (mm Hg)∆	0.036	0.727	–	–	
eGFR (mL/min) ∆	0.093	0.313	–	–	
LV remodeling index	− 0.270	0.000	–	–	

*BMI* body mass index, *eGFR* estimated glomerular filtration rate, *HDL* high-density lipoprotein, *SBP* systolic blood pressure, *TG* plasma triglycerides

^a^Candidate variables for multivariable model were selected on clinical grounds, guided by univariable correlation with P value < 0.10 and the absence of collinearity. ^∆^Age, SBP, and eGFR was included in the multivariable analysis model based on clinical grounds. Medication history was included in the multivariable model but not listed because there was no significant correlation in univariable analysis

### The ROC curve analysis of individuals on global upslope in the same BMI scale for discriminating the additive effect of T2DM status

**Fig. 4:**
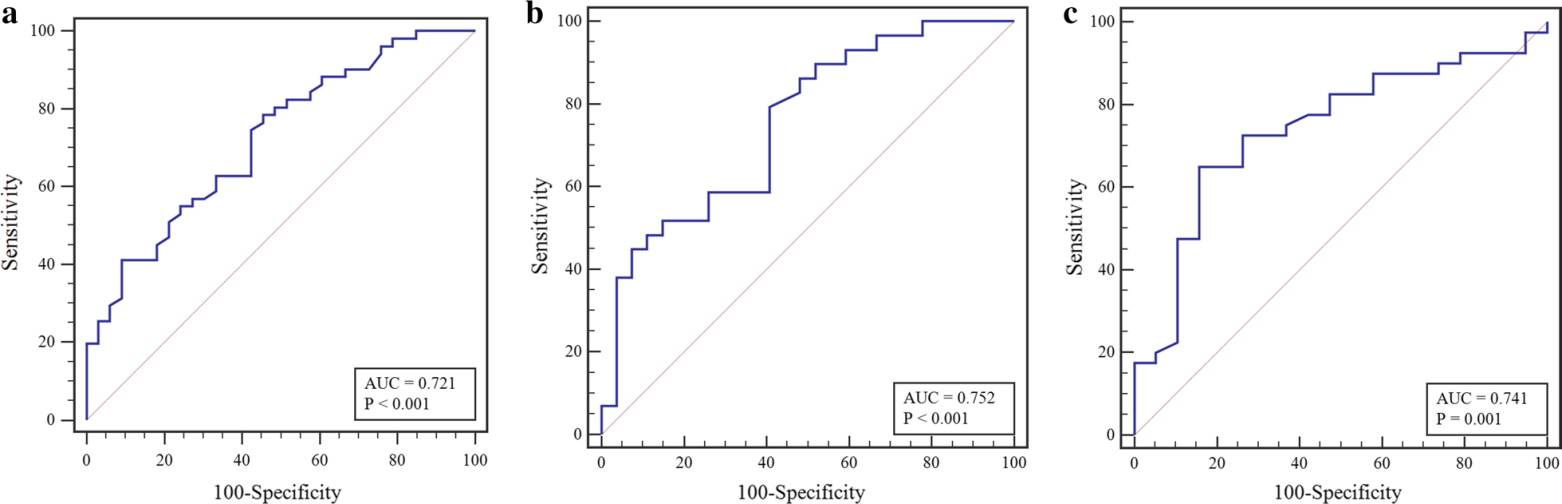
The ROC curve analysis of global upslope between patients with T2DM and controls with comparable BMIs (**a**, normal-weight group; **b**, overweight group; obese group)

The area under the curve (AUC), sensitivity, ﻿specificity and optimal cut-off value were determined by the ROC curve analysis to assess the additive effect of T2DM on global upslope by comparison with the controls in the same BMI scale. As shown in Fig. 4, the CMR first-pass imaging had a moderate efficiency in identifying the additive effect of the T2DM status in all BMI scales (normal-weight group: AUC 0.721, sensitivity 54.5%, specificity 78.4%, cut-off value 2.81, *P* = 0.001; overweight group: AUC 0.752, sensitivity 59.3%, specificity 79.3%, cut-off value 2.26, *P* = 0.001; obese group: AUC 0.741, sensitivity 84.2%, specificity 65.0%, cut-off value 1.45, *P* = 0.003).

### The logistic regression analysis for independent predictors of myocardial microvascular dysfunction

The study cohort was categorised into two groups based on the optimal cut-off value determined by the ROC curve in different BMI scales, and the results of the binary univariable and multivariable logistic regression analyses are shown in Table 4. The multivariable logistic regression analysis indicated that T2DM was an independent predictor of microvascular dysfunction in normal-weight (odds ratio[OR], 6.46; 95% CI, 2.08 to 20.10; *P* = 0.001), overweight (OR, 7.19; 95% CI, 1.67 to 31.07; *P* = 0.008) and obese participants (OR, 11.21; 95% CI, 2.38 to 52.75; *P* = 0.002) and that the OR increased with increasing BMI.

**Table 4:** Univariable and multivariable logistic regression analysis in independent predictors of myocardial microvascular dysfunction

	Normal–weight group		Overweight group		Obese group	
	Univariable OR (95% CI)	Multivariable OR (95% CI)	Univariable OR (95% CI)	Multivariable OR (95% CI)	Univariable OR (95% CI)	Multivariable OR (95%CI)
Sex (female)	0.40 (0.16–0.99)^∆^	0.20 (0.06–0.61)^†^	0.10 (0.03–0.34)^†^	0.08 (0.02–0.34)^†^	0.17 (0.05–0.54)^†^	0.12 (0.03–0.47)^†^
Age	1.01 (0.97–1.05)	–	1.03 (0.97–1.08)	–	0.99 (0.94–1.04)	–
Diabetes	3.90 (1.52–10.01)^†^	6.46 (2.08–20.10)^†^	5.58 (1.71–18.18)^†^	7.19 (1.67–31.07)^†^	8.00 (2.00–31.99)^†^	11.21 (2.38–52.75)^†^
Hypertension	3.25 (0.99–10.74)	–	3.03 (0.74–12.45)	–	1.26 (0.40–3.99)	–
Hyperlipidemia	9.20 (1.14–74.25)*	–	0.96 (0.28–3.22)	–	0.44 (0.12–1.65)	–
Renal dysfunction^^^	1.75 (0.33–9.27)	–	4.50 (0.50–40.26)	–	10.85 (1.24–94.97) *	–

Candidate variables for multivariable model were selected on clinical grounds, guided by univariable correlation with P value < 0.10 and the absence of collinearity. ^**^**^Renal dysfunction: estimated creatinine clearance < 60 mL/min

∆P < 0.1; *P < 0.05; ^†^P < 0.01. *CI* confidence interval, *OR* odds ratio

### Intra-observer and inter-observer variability

The intra-observer and inter-observer correlation coefficients were considered excellent, and summarized in Additional file 1: Table S1.

## Discussion

In the current study, we analysed microvascular perfusion in patients with T2DM and nondiabetic controls with different BMI scales who were assessed by CMR first-pass perfusion imaging. We found that myocardial microvascular function exhibited a gradual decline with increasing BMI in both the controls and the patients with T2DM, and the patients with T2DM had worse resting microvascular function compared with the controls within the same BMI scale. Further logistic regression analysis indicated that T2DM was an independent predictor of microvascular dysfunction in all participants and that the risk was higher in obese individuals. Overall, these findings indicate that T2DM and obesity may cause myocardial microvascular dysfunction even in the resting state, and that obesity might exacerbate the adverse effects of DM on microvascular function.

### The combined effects of obesity and diabetes on myocardial microvascular damage

Obesity- and T2DM-related cardiac dysfunction have shared underlying disease mechanisms as both are metabolic disorders; despite the complex and multifactorial pathophysiology of cardiomyopathy related to obesity and T2DM, alterations in cardiac energy metabolism and subsequently energetics are recognised as major contributors to cardiac dysfunction in both conditions [19, 20]. As a result of metabolic and energy disorders, both structure and function abnormalities are associated with coronary microvascular dysfunction [21]. A previous study suggested that concomitant diabetes and obesity may exacerbate microvascular injury [22]. In the this prospective clinical study, consistent with the existing mechanism above, we demonstrated not only that T2DM or obesity alone can cause myocardial microvascular dysfunction, but also that the obesity status exacerbates the adverse effect of T2DM on microcirculatory damage.

### The effects of obesity and diabetes alone on myocardial microvascular damage

Regarding the effects of obesity on myocardial microvascular function, Schindler et al. found that a high BMI was independently associated with abnormal microvascular function based on the gradual impairment of vasodilation capacity with increasing BMI [23]. In agreement with that study, we found that there was a gradual decline in microvascular function with increasing BMI in all participants. These results might be attributable to the potential impairment of the endothelium-related coronary vasomotion or microcirculatory structure by obesity, even under resting conditions. Nevertheless, these potential mechanisms require further investigation. Moreover, our results indicated that the LV remodelling index was more pronounced in the study subjects with a higher BMI. The LV remodelling, the main pathology underlying chronic heart failure and an important prognostic indicator [24, 25], was previously reported to develop as a direct result of high metabolic activity and lipotoxicity in obese individuals [16].

Diabetic cardiomyopathy is a complex condition with multiple involved pathways and multiple mechanisms, and various factors interact to cause functional and vascular changes in the heart [26]. Previous studies have reported obesity, hyperglycaemia, high HbA1c levels and a longer diabetes duration as risk factors for the progression of diabetic cardiomyopathy [27, 28]. Coronary microvascular dysfunction as a direct cause of myocardial tissue hypoxia is an important factor involved in the development of diabetic cardiomyopathy. In this study, consistent with some of the reported risk factors for diabetic cardiomyopathy, microvascular dysfunction was more likely to be affected by sex, BMI, diabetes duration and HbA1c levels, among which BMI is the dominating factor. We speculate that the aforementioned mechanisms of microcirculatory damage shared between obesity and diabetes underlie the dominant adverse role of BMI in microvascular dysfunction in patients with T2DM. Among the risk factors for microvascular dysfunction that were identified in the current study, HbA1c, an indicator of blood glucose control in patients with DM, was a unique risk factor in the patients with T2DM that was independently associated with myocardial microvascular function. This finding further supports the possibility that intensive glycaemic control may be beneficial for preserving myocardial microcirculation [29, 30].

### Other related factors affecting myocardial microcirculation

Glucose-lowering drugs are a fundamental strategy for T2DM management. Currently, multiple pharmacological agents are available for controlling glycaemia, which is selected to be individualised according to each patient’s needs. Several studies have indicated the relationship between hypoglycaemic agents and myocardial microcirculation and cardiovascular events, but the benefits and possible adverse effects of some medications for patients with diabetes, especially in those with heart failure, remain controversial [31–33]. In our study, multiple drugs were recorded and included in the analysis, indicating that the drug type had no effect on myocardial microcirculation. Further randomised controlled trials are needed to determine whether the drug combination interferes with the results.

﻿Sexual dimorphism is a very well-established and common characteristic of cardiovascular disease, and previous studies have indicated that both diabetic cardiomyopathy and heart failure with preserved ejection fraction are more prevalent in females than in males [34, 35]. However, in the current study, females with diabetes were less likely to present with myocardial microvascular dysfunction, and female sex was a protective factor for microcirculation dysfunction. This inconsistency may be due to the potential protective effect of oestrogen against the apoptosis and necrosis of endothelial cells in microcirculation [36].

Consistent with the report on cardiac amyloid light-chain amyloidosis by Li et al. [12], among the myocardial perfusion parameters, a gradient of basoapical increase was observed for upslope, MaxSI, MaxSI (-BL) and SI (baseline) in both the patients with T2DM and the controls. Furthermore, T2DM and obesity presented lower parameter values compared with the corresponding controls. Based on the current study results and the previously published data, we speculate that the LV might undergo a gradual variation in organisational structure, metabolite deposition and microvascular density distribution from the base to the apex, and that the apex may have higher resistance to microvascular injury. Several metabolic substances and pathological changes have been examined using CMR imaging, and further confirmation will be necessary in future studies.

### Limitations

The current study has several limitations. First, this was a single-centre study and all participants were evaluated using one CMR scanner; therefore, the reference values may be different than those used with other scanners. However, utilising the same CMR scanner eliminates differences in results that might arise from the use of different scanners. Multi-centre and multivendor studies should be planned to address this concern. Second, because of the contraindications and potential risks associated with cardiac stress testing [37, 38], this study investigated microcirculation function only at rest. However, even in the resting state, our study demonstrates the additive effect of obesity on myocardial microcirculation in diabetic individuals, and the clinical value of resting myocardial perfusion examination requires more attention and verification. Third, our study did not include patients with T2DM who had a low body weight (BMI < 18 kg/m^2^); the metabolic mechanisms of these individuals differ from those in patients with a higher body weight. Finally, not all participants underwent coronary computed tomographic angiography or invasive coronary angiography to exclude coronary artery disease in the current study. In the present study cohort, coronary artery disease was deemed to be unlikely based on the assessment of the subjects by electrocardiography, echocardiography, laboratory examination and clinical history, further supported by the subsequent CMR examinations.

## Conclusions

There was a gradual decrease in myocardial microvascular function with increasing BMI in both the patients with T2DM and the nondiabetic controls, even under resting conditions. When T2DM and obesity coexist, T2DM increased the risk of microvascular dysfunction, and the obesity status exacerbated the effects of T2DM. These findings demonstrate the additive effect of obesity on myocardial microcirculation in diabetic patients and highlight the importance of weight loss in T2DM individuals with obesity.

## Supplementary information

**Additional file 1: Table S1.** Intra-observer and Inter-observer variability of perfusion parameters.

## Data Availability

The datasets used and analyzed during the current study are available from the corresponding author on reasonable request.

## Abbreviations

BMI: Body mass index
CMR: Cardiac magnetic resonance
ECV: Extracellular volume fraction
EDV: End-diastolic volume
EF: Ejection fraction
ESV: End-systolic volume
HbA1c: Glycated hemoglobin
LV: Left ventricular
MaxSI: Max signal intensity
ROC: Receiver operator characteristic
T2DM: Type 2 diabetes mellitus
TTM: Time to maximum signal intensity
