# Supplementary material for: The additive effects of obesity on myocardial microcirculation in diabetic individuals: a cardiac magnetic resonance first-pass perfusion study
Source: Cardiovasc Diabetol. 2020 May 6;19:52. doi: 10.1186/s12933-020-01028-1 (PMC7201945; doi:10.1186/s12933-020-01028-1)
Supplement: Supplementary file 1 — Additional file 1: Table S1. Intra-observer and Inter-observer variability of perfusion parameters. [file 12933_2020_1028_MOESM1_ESM.docx]

**Table S1 Intra-observer and Inter-observer variability of perfusion parameters**

|  | **Intra-observer(n=60)** | |  | **Inter-observer(n=55)** | |
| --- | --- | --- | --- | --- | --- |
|  | ICC | 95% CI |  | ICC | 95% CI |
| ***Basal*** |  |  |  |  |  |
| Upslope | 0.988 | 0.965-0.996 |  | 0.996 | 0.991-0.998 |
| TTM (sec) | 0.863 | 0.651-0.892 |  | 0.799 | 0.542-0.877 |
| MaxSI | 0.931 | 0.896-0.980 |  | 0.958 | 0.913-0.980 |
| MaxSI (- baseline) | 0.955 | 0.930-0.984 |  | 0.989 | 0.977-0.995 |
| SI(Baseline) | 0.976 | 0.953-0.987 |  | 0.982 | 0.961-0.991 |
| ***Mid-ventricular*** |  |  |  |  |  |
| Upslope | 0.946 | 0.921-0.987 |  | 0.990 | 0.979-0.995 |
| TTM (sec) | 0.912 | 0.897-0.951 |  | 0.979 | 0.955-0.990 |
| MaxSI | 0.921 | 0.908-0.972 |  | 0.978 | 0.953-0.989 |
| MaxSI (- baseline) | 0.954 | 0.931-0.979 |  | 0.979 | 0.957-0.990 |
| SI(Baseline) | 0.959 | 0.932-0.981 |  | 0.976 | 0.949-0.989 |
| ***Apex*** |  |  |  |  |  |
| Upslope | 0.882 | 0.832-0.927 |  | 0.899 | 0.854-0.913 |
| TTM (sec) | 0.801 | 0.769-0.853 |  | 0.755 | 0.736-0.847 |
| MaxSI | 0.887 | 0.856-0.924 |  | 0.911 | 0.882-0.938 |
| MaxSI (- baseline) | 0.919 | 0.886-0.949 |  | 0.923 | 0.899-0.957 |
| SI(Baseline) | 0.901 | 0.872-0.928 |  | 0.918 | 0.883-0.939 |

The intra-observer variability was 0.801–0.988, while the inter-observer variability was 0.755–0.996. The intra-observer and inter-observer correlation coefficients were considered excellent for all parameters, except TTM in the apical slice (inter-observer correlation coefficient 0.755).
